# A dual-stream temporal attention LSTM with visual feature enhancement for depression screening in incarcerated populations

**DOI:** 10.3389/fpsyg.2026.1850264

**Published:** 2026-06-10

**Authors:** Zhifei Xu, Shiyun Shao, Jiagang Dong, Jiafu Zhou, Yihan Zeng, Yichao Zhang

**Affiliations:** 1 Zhejiang Provincial Academy of Judicial Administration, Zhejiang Police Vocational Academy, Hangzhou, China; 2 Zhejiang Provincial Engineering Research Center for Brain Cognition, Disease and Digital Medical Devices, School of Information Engineering, Hangzhou Medical College, Hangzhou, China; 3 Department of Digital Technology, Zhejiang Yuying College of Vocational Technology, Hangzhou, China

**Keywords:** depression screening, dual-stream LSTM, efficient local attention, facial action units, facial expression recognition, incarcerated individuals, temporal attention

## Abstract

**Background/objective:**

Incarcerated individuals face high depression risk due to the stressful closed correctional environment. Facial expressions best reflect emotional and psychological states, but traditional screening methods are subjective and fail to capture their temporal dynamics, so this study proposes an automated screening method based on visual temporal modeling.

**Methods:**

A Dual-Stream Temporal Attention LSTM (DSTA-LSTM) model was developed for depression screening. Seventy-six incarcerated participants provided 1,216 valid video segments, which were preprocessed via semantic chunking and optical flow enhancement to capture subtle micro-expression dynamics. Visual features were extracted via MobileNetV2 with an Efficient Local Attention (ELA) module, and physiological features via 20 facial Action Units (AUs) and valence-arousal vectors. Dual LSTM branches modeled temporal features of both streams, which were fused by an attention mechanism for accurate depression screening, and model performance was evaluated via 5-fold cross-validation, ablation experiments, and SHAP analysis.

**Results:**

The DSTA-LSTM significantly outperformed five baseline models, including ResNet50, 3D-CNN, and TimeSformer, achieving an AUC of 0.934, F1-score of 0.892, sensitivity of 0.913, and specificity of 0.886. Ablation experiments showed that removing the ELA module, AU stream, or temporal attention reduced the AUC by 5.1, 2.6, and 1.7%, respectively. SHAP analysis revealed AU4 and AU15 as the most influential features, which is consistent with clinical depression manifestations.

**Conclusion:**

The DSTA-LSTM has strong visual detection capability for facial dynamics, offering a reliable automated solution for incarcerated depression screening and important significance for public health and security; future research will integrate multimodal data to further improve model performance.

## 1 Introduction

Depression has become one of the most common mental disorders worldwide (Smith, 2014), and its main characteristic is persistent low mood. This issue is particularly severe in correctional facilities including prisons and detention centers. Its clinical presentations include persistent sadness, anhedonia, fatigue, and a variety of somatic and cognitive symptoms (Park et al., 2019). As a high-risk group, incarcerated individuals face a significantly higher prevalence of depression due to the special stressors of correctional settings, making targeted and efficient depression screening particularly urgent for this population.

Despite being required to adhere to institutional rules and standardized codes of conduct such as the Prison Inmates’ Code of Conduct, incarcerated individuals originate from highly heterogeneous social backgrounds and exhibit substantial variability in baseline mental health status. This closed and restrictive correctional environment with considerable stress may further worsen latent psychological vulnerabilities or even trigger the development of mental health disorders (Goomany and Dickinson, 2015). As reported by Wainwright et al., nearly half of the incarcerated population presents clinically significant mental health problems (Baranyi et al., 2022). Standard assessments employing the Symptom Checklist 90 (SCL-90) scale indicate that depression factor scores among incarcerated individuals are significantly higher than national normative data for the general population (Juarros-Basterretxea et al., 2021). Importantly, those with pre-existing mental health conditions or newly onset psychological symptoms in the correctional setting exhibit a markedly elevated risk of recidivism (Baker et al., 2023). Delayed or insufficient depression screening increases the likelihood of self-harm and suicidal behaviors among incarcerated individuals. It also escalates security risks within correctional institutions. In severe cases, it threatens broader public safety upon community reentry.

Nevertheless,depression screening among incarcerated populations faces substantial practical and methodological challenges. Psychological risk assessment scales are the primary instruments for psychological evaluations of incarcerated individuals (Preti et al., 2019) and the most commonly used tools include the Symptom Checklist 90 (SCL-90), the Chinese Criminal Psychological Test Personality Inventory (CAPO-PI), the Eysenck Personality Questionnaire (EPQ), and the Cattell 16 Personality Factor Questionnaire. However, as the number of incarcerated individuals continues to grow, the limitations of these scale-based assessments have become increasingly apparent. Many incarcerated individuals have low educational attainment, with over 60% of inmates having an education level below junior high school (McCarron et al., 2021), which leads to significant misunderstandings of scale questions. Additionally, some prisoners may deliberately falsify answers out of concern for affecting their parole chances (Gudjonsson et al., 2021), and scale assessments cannot capture temporal fluctuations in inmates’ emotional states (Jeon et al., 2025). While combining scale assessments with clinical interviews can yield more accurate data, clinical interviews are hindered by a shortage of specialized professionals. According to 2022 data from the Ministry of Justice, the ratio of psychologists to incarcerated individuals in correctional facilities is only 1:500, with each interview taking no less than 30 min and the daily screening capacity being fewer than 30 people. Furthermore, different clinicians may be influenced by their personal experience and subjective biases, resulting in inconsistent evaluative judgments (Ko et al., 2025).

Related studies have shown that changes in human emotional and psychological states can lead to comprehensive alterations in facial expressions, speech, body posture, and physiological signals. Such behavioral and physiological variations can reflect different emotional and psychological states to varying degrees (Barrett et al., 2019). According to Mehrabian’s 7–38-55 communication rule, 55% of emotional information is conveyed through visual channels, 38% through vocal channels, and only 7% through verbal content (Amsel, 2019). This theoretical framework underlines the great significance of facial expressions for objective emotion recognition and mental state evaluation.

Given the key limitations of traditional depression screening approaches and the strong demand for efficient screening among incarcerated populations, this study proposes a depression screening method for this group based on facial expression recognition: Dual-Stream Temporal Attention LSTM with Visual Feature Enhancement. This method is designed to reliably capture emotional traits and address the flaws of traditional screening scales.

## 2 Related work

Current research on facial expression-based depression screening models has two primary technical paths. They involve single static image recognition and image-driven multimodal data collection and recognition. These two methods differ greatly in data collection difficulty and application scenarios, with their own advantages and disadvantages.

The first approach focuses on recognition from single static images. The advantages of this approach include high computational efficiency. For example, Kim et al. (2023) studied a depression expression recognition model based on the Facial Action Coding System (FACS), achieving depression screening by analyzing the static combinations of AU (action units). Their ResNet-50 model processed each image in just 15-30 ms (Contardo et al., 2023). Additionally, publicly available datasets, such as FER-2013, which contains 35,887 static expression images, enable quick transfer learning training (Goodfellow et al., 2015). In terms of leveraging large datasets to improve the generalization ability and accuracy of algorithms, Valstar et al. (2014) combined spatial texture features with Gabor filtering to obtain facial visual features for depression recognition. Verma et al. (2020) proposed the lateral growth mixed network (LEARNet) to capture micro-features of facial regions, achieving good results. However, this approach has significant drawbacks: it fails to leverage the temporal features of facial expressions, as a single image cannot capture continuous or micro-expression changes, and it is susceptible to disguised expressions—Roy and Strate (2023) noted that incarcerated individuals could manipulate their facial expressions to avoid screening, while Klingner and Guntinas-Lichius (2023) found single images insufficient for capturing complex emotions, reducing recognition accuracy.

The second approach focuses on image-driven multimodal data collection and recognition. This approach improves recognition performance by integrating video and other physiological signals, with the core breakthrough being the joint modeling of spatial and temporal features. For example, Jinlong Hu et al. proposed the SFEM module, which uses a two-level temporal modeling approach that integrates sparse sampling and attention mechanisms. This method reached a cross-dataset accuracy of 87.3% in dynamic expression analysis (Hu et al., 2024). Although accuracy has been improved, it still has several key limitations. Data Synchronization: Video and physiological signals use different sampling rates, which leads to feature misalignment and biased decisions. Training complex fusion models also demands heavy resources and high costs. Deployment Difficulties: High latency and model complexity make it hard to deploy in correctional facilities, as laboratory conditions differ greatly from real correctional environments (Samadiani et al., 2019). In addition, some solutions depend on contact-type EEG devices (Cruz-Gonzalez et al., 2025). Privacy and Ethical Concerns: Privacy risks are significant, with related breaches and ethical dilemmas documented (Malek et al., 2023). Notably, multimodal data still depend heavily on image-based analysis (Zhao et al., 2016), yet their equipment costs are significantly higher than those of single-modal methods (Wang et al., 2016).

Moreover, existing research has key unsolved problems that correspond with the main challenges of this study. Incarcerated people often deliberately hide their true feelings, so disguised emotional expressions are a critical issue. Current detection models lack stability to reliably spot these behaviors in correctional facilities. This work therefore focuses on improving micro-expression capture capability (Jiang et al., 2014). Also, some methods fail to structure long video data properly, fragmenting emotional context. Others lack efficient lightweight feature extraction. Multimodal approaches are costly and hard to deploy in practice. These gaps call for dynamic temporal and micro-expression analysis, plus targeted preprocessing and lightweight network design to enable reliable depression screening in correctional settings.

In summary, this study addresses limitations of traditional depression screening in correctional settings—subjectivity, vulnerability to emotional camouflage, and weak temporal modeling. To fix these, we propose a Dual-Stream Temporal Attention LSTM (DSTA-LSTM). This work has three main contributions:

1. A data preprocessing strategy based on semantic chunking and motion feature enhancement is developed, which structures long video sequences and strengthens the model’s capability to capture spatiotemporal features of facial micro-expressions;
2. A lightweight feature extraction network (MobileNetV2-ELA) embedded with Efficient Local Attention (ELA) is designed, enhancing the recognition accuracy of depression-related facial representations while maintaining computational efficiency;
3. A Dual-Stream temporal attention LSTM screening model that fuses visual and physiological cues is constructed, integrating deep visual features and facial action unit dynamics to realize robust and accurate automated depression screening.

## 3 Materials and methods

### 3.1 Overview of the research framework

**Figure 1:**
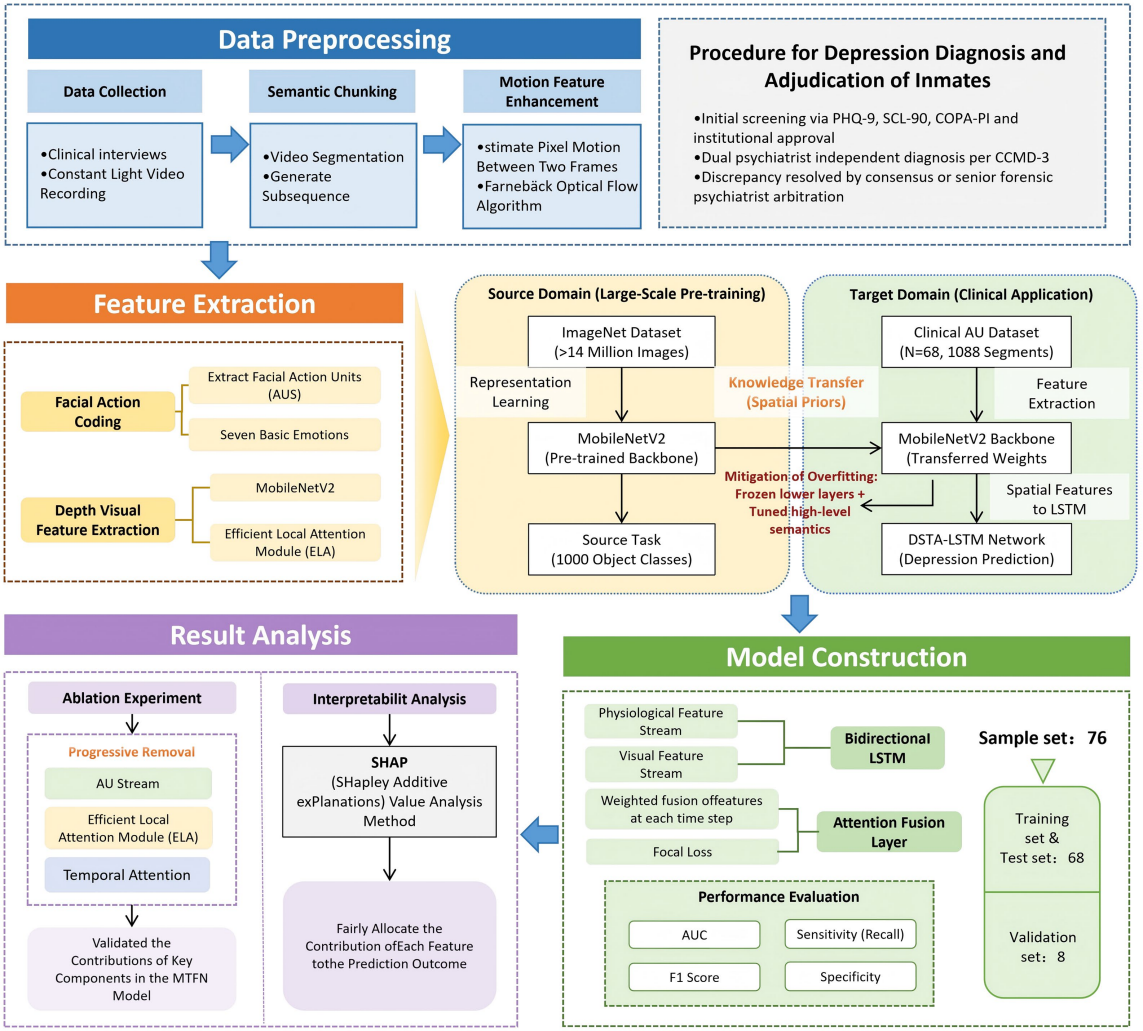
Model framework diagram.

This study proposes a dual-stream temporal attention long short-term memory network (DSTA-LSTM) for depression screening among the incarcerated population based on the temporal expression dynamic model. The overall workflow is detailed in Figure 1: First, data are collected from clinical interview videos, followed by image preprocessing, which involves semantic segmentation first and then feature enhancement. Subsequently, multi-dimensional facial features are extracted and used to model temporal dynamics and frame-level detailed features. These features are then input into the DSTA-LSTM architecture. A dedicated attention fusion layer in the network effectively integrates features from different modalities. To objectively evaluate the model’s performance, indicators such as sensitivity, specificity, F1 score, and AUC are used in this paper. At the same time, rigorous ablation experiments and interpretability analyses are conducted to validate our research findings. The subsequent sections will provide a systematic elaboration on this.

### 3.2 Data preparation

In this study, 76 participants were recruited from a correctional institution in eastern China, and a primary dataset consisting of 1,216 video clips of clinical interviews was constructed. Among the participants, 49 were male (64.5%) and 27 were female (35.5%), with an average age of 39.4 years. There were significant differences in their educational levels, ranging from no formal education (1.3%) to having a university degree (30.3%). The vast majority of the samples(93.4%) were first-time offenders with no prior criminal records. Regarding the prison terms at that time, most participants were imprisoned for 1–3 years (63.3%), and the overall recidivism rate of the entire sample was 27.6%. More importantly, all participants were informed of the research purpose, procedures, and potential risks before participating in the study, and they all signed a formal written informed consent form.

Three types of emotion-induction tasks were designed based on standardized psychological paradigms. Firstly, the picture-induced emotional description task: pleasant, sad and neutral facial expression images were sequentially presented on a screen, with the stimulus materials derived from the Chinese Facial Affect Picture System (CFAPS) and the Chinese Affective Picture System (CAPS); participants were required to briefly describe their emotional responses after viewing each image. Secondly, the structured interview task: the interview outline was compiled by integrating items from the Symptom Checklist-90 (SCL-90), Patient Health Questionnaire-9 (PHQ-9), Hamilton Depression Rating Scale (HAMD), Beck Depression Inventory (BDI) and the Diagnostic and Statistical Manual of Mental Disorders (DSM), consisting of 10 questions (2 positive, 3 neutral and 5 negative ones), and participants answered the questions as instructed. Thirdly, the text reading task: participants were asked to read three segments of text materials with a balanced distribution of positive, neutral and negative emotional words. Details of the test protocol are provided in the supplementary document.

Diagnoses of depression among incarcerated individuals were established by two board-certified psychiatrists each with more than 5 years of clinical experience, in accordance with the Chinese Classification and Diagnostic Criteria of Mental Disorders (3rd Edition) (CCMD-3) (Dai et al., 2014), which is consistent with the diagnostic criteria of the International Classification of Diseases (ICD-10). We adopted a standard arbitration procedure to ensure objectivity and resolve any diagnostic disagreements. Any disagreement between the two psychiatrists was first addressed through a structured consensus discussion. If they could not reach an agreement, a third senior psychiatrist was invited to provide a final verdict. This expert had over 10 years of experience in forensic psychiatry. This process ensured that the diagnostic labels used for model training and evaluation were reliable.

Video data were collected during clinical interviews in a constant illumination environment. To ensure data quality, participants were instructed to maintain a natural state during the interviews and avoid deliberately controlling their facial expressions.

To strictly prevent data leakage, the dataset was partitioned at the subject-ID level. Specifically, 68 of the 76 participants were allocated to model development, undergoing five-fold cross-validation. The remaining 8 participants (comprising 124 video segments) were reserved as a held-out test set, strictly utilized only once for the final performance evaluation.

The study protocol was approved by the Institutional Review Board of Zhejiang Police Vocational Academy (Approval no. 21G010006; May 12, 2021) and conducted in strict adherence to the Declaration of Helsinki and relevant medical research guidelines. All participant data were entirely anonymized. Given the specific nature of the cohort, individuals were explicitly assured that their involvement was strictly voluntary, and that refusing to participate or withdrawing from the study at any point would have absolutely no bearing on their legal status, parole eligibility, or conditions of confinement.

### 3.3 Data preprocessing

#### 3.3.1 Semantic segmentation processing

To effectively capture facial expression changes related to depression, this paper divides the video into discrete semantic blocks according to the timestamps of interview questions, while retaining the corresponding text labels for subsequent analysis. For example, the video segment corresponding to the prompt question “Have you felt depressed in the past 2 weeks?” will be regarded as an independent semantic unit. Additionally, to ensure that no key information is missed, three subsequences will be extracted from each question block. The above segmentation strategy divides the video into multiple segments with different semantic contexts, facilitating structured feature extraction and analysis.

#### 3.3.2 Motion feature enhancement

In the context of depression screening, subtle changes in facial expressions are key indicators of potential emotional states. To capture these dynamic changes with high fidelity, this study employs the Farnebäck optical flow algorithm to compute the dense optical flow field. As a computational technique based on polynomial expansion, this algorithm can effectively estimate the local spatial transformation between consecutive frames. To introduce this pure motion information into the deep learning model, we convert the computed optical flow field into independent motion feature maps and input them into parallel network branches as additional input channels. The optical flow field explicitly encodes the motion trajectories of pixel intensities, thereby effectively enhancing the model’s ability to extract spatio-temporal features of micro-expressions. The specific computational procedure is detailed as follows:

First, two consecutive frames are converted to grayscale images, with the conversion formula as shown in Equation 1:


$$I_{g\mathrm{ray}}=0.299\cdot R+0.587\cdot G+0.114\cdot B\;\;$$ (1)

Where R, G, and B represent the red, green, and blue channel values of the image, respectively.

The Farnebäck algorithm approximates the local neighborhood of each pixel through a second-order polynomial expansion. For a pixel at position (x, y), the grayscale value I(x, y) within its neighborhood can be approximated as shown in Equation 2:


$$I(x,y)\approx x^{T}\mathrm{Ax}+b^{T}x+c\;\;$$ (2)

Where x = [*x,y*]*^T^* is the pixel coordinate, A is a 2 × 2 symmetric matrix, *b* is a 2 × 1 vector, and c is a scalar constant.

Assuming the optical flow vector between the two frames is *d =* [*dx,dy*]*^T^*, the relationship between the two frames can be expressed as shown in Equation 3:


$$I_{1}(x)=I_{2}(x+d)\;\;\;$$ (3)

Performing a first-order Taylor expansion of *I_2_*(*x + d*) and neglecting higher-order terms yields Equation 4:


$$I_{2}(x+d)\approx I_{2}(x)+\nabla I_{2}{\left(x\right)}^{T}d\;\;$$ (4)

Where*𝛻I_2_*(*x*) is the gradient of *I_2_* at x.

By substituting the polynomial expansion into the optical flow constraint, the optical flow vector d is solved using the least squares method, as shown in Equation 5:


$$d={\left(A_{1}-A_{2}\right)}^{-1}(b_{2}-b_{1})\;\;$$ (5)

The computed optical flow field d is decomposed into horizontal (*d_x_*) and vertical (*d_y_*) components, as defined in Equation 6:


$$d_{x}=d[\theta ],d_{y}=d[1]\;\;$$ (6)

To ensure input data consistency, therefore, both components are normalized to the range [−1, 1], as shown in Equation 7:


$$d_{x}^{\mathrm{nom}}=\frac{d_{x}-\min(d_{x})}{\max(d_{x})-\min(d_{x})},\;\;d_{y}^{\mathrm{nom}}=\frac{d_{y}-\min(d_{y})}{\max(d_{y})-\min(d_{y})}\;\;$$ (7)

**Figure 2:**
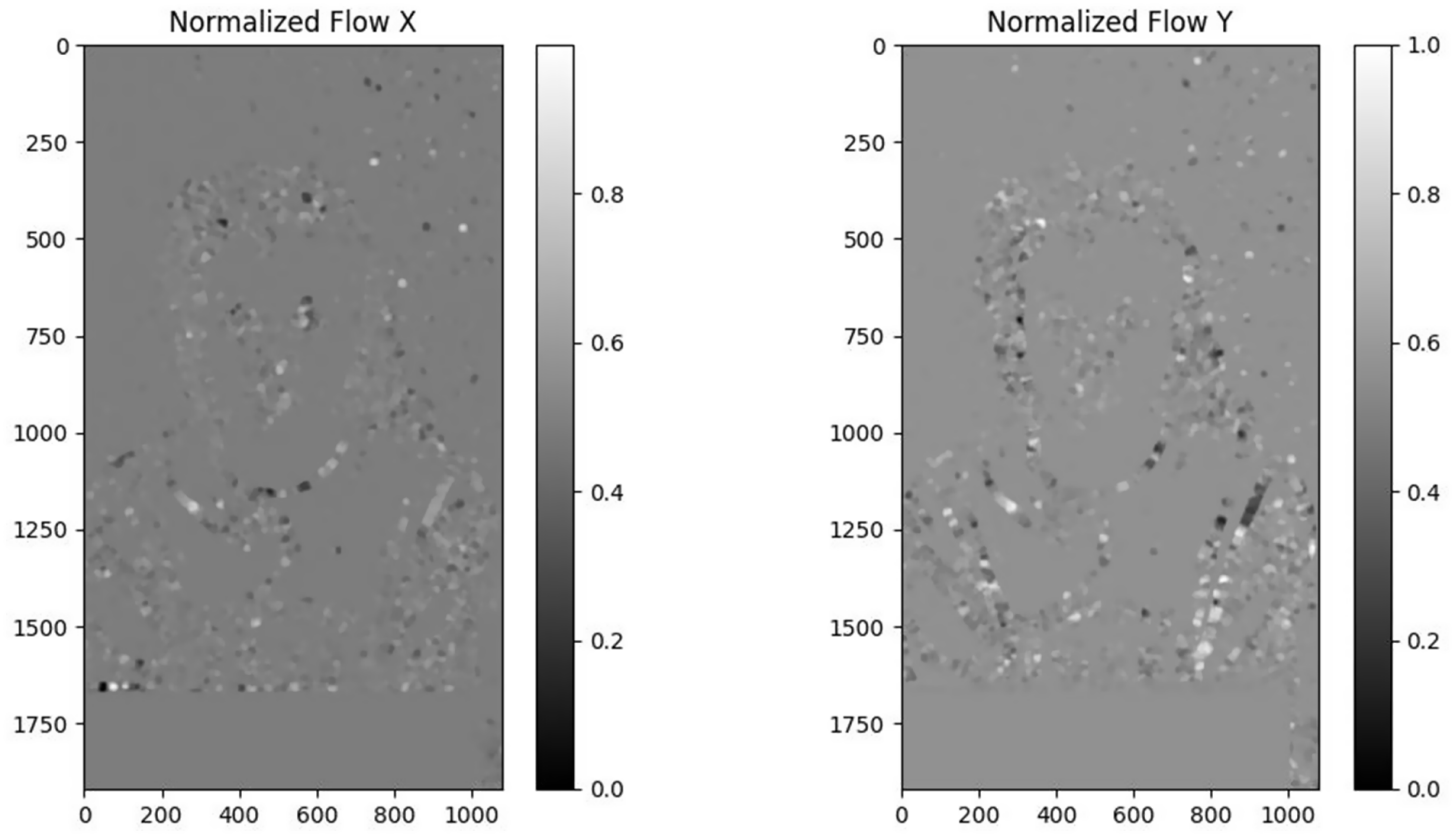
Normalized optical flow components diagram.

The normalized optical flow components dxnom and dynom are used as additional input channels (as shown in Figure 2) and are fed into the subsequent feature extraction network along with the original image data.

### 3.4 Facial expression feature engineering

Depressed individuals manifest distinct and observable alterations in facial expressions, which are intrinsically linked to their underlying physiological characteristics (Sadeghi et al., 2024). To comprehensively characterize these facial expression states, this study employs a multi-source feature extraction framework that synergistically integrates visual and physiological features.

#### 3.4.1 Facial AUs and emotion extraction

This study used py-feat (v0.6.2) to extract 20 facial Action Units (AUs), including AU01, AU02, AU04, AU05, AU06, AU07, AU09, AU10, AU11, AU12, AU14, AU15, AU17, AU20, AU23, AU24, AU25, AU26, AU28 and AU43, with details summarized in Table 1. Facial Action Units (AUs) represent the fundamental components of facial muscle movements. By capturing subtle facial dynamics, AU extraction enhances the precision of identifying depression-related expression features.

**Table 1:** Emotions to AUs mapping.

AU number	FACS name	Associated emotions
AU01	Inner brow raiser	Key features of sadness; fear
AU02	Outer brow raiser	Surprise; fear
AU04	Brow lowerer	Core negative emotions; depression
AU05	Upper lid raiser	Surprise; fear; anger (wide eyes)
AU06	Cheek raiser	Happiness (smile); sad pain
AU07	Lid tightener	Anger; focus; suspicion
AU09	Nose wrinkler	Disgust
AU10	Upper lip raiser	Disgust; anger; sadness (twitch)
AU11	Nasolabial deepener	Sadness; deep anxiety
AU12	Lip corner puller	Happiness
AU14	Dimpler	Contempt; forced smile
AU15	Lip corner depressor	Direct sadness indicator
AU17	Chin raiser	Sadness; disgust; anger (chin creases)
AU20	Lip stretcher	Fear (panic mouth movement)
AU23	Lip tightener	Anger; restraint; endurance
AU24	Lip pressor	Anger; focus; controlled anger
AU25	Lips part	Intense emotions (speak/shout/laugh)
AU26	Jaw drop	Surprise
AU28	Lip suck	Anxiety; restraint; cry suppression
AU43	Eyes closed	Sadness; fatigue; eye avoidance

Furthermore, discrete emotions are mapped onto the valence-arousal two-dimensional space to quantify emotional states. Valence denotes the positive or negative quality of an emotion, while arousal measures the intensity of the emotional response. These two dimensions constitute a 2D framework for emotional quantification, with the corresponding calculation formulas presented in Equation 8:


$$V=\sum_{i=1}^{7}w_{i}^{\nu }e_{i},\;\;A=\sum_{i=1}^{7}w_{i}^{a}e_{i}\;\;$$ (8)

Here, *V* and *A* stand for valence and arousal, respectively. $w_{i}^{\nu }$ and $w_{i}^{a}$ are the valence and arousal weights assigned to each emotion, and $e_{i}$ denotes the intensity of that emotion. This mapping captures emotional states more fully and delivers more detailed information for later feature fusion steps.

Since the widespread facial occlusion and variations in lighting conditions in the correctional environment can significantly affect the stability and reliability of Action Unit (AU) detection, to systematically address this issue, this study used the Py-Feat framework to quantify the confidence level of each extracted AU, resulting in an overall average confidence level of 0.86. To ensure the temporal continuity of subsequent optical flow analysis and LSTM modeling, we set a confidence threshold of0.80 during the data pre-processing stage. For low-confidence segments with a duration of less than 0.5 s, a linear interpolation algorithm of adjacent high-confidence frames was used for data repair. Meanwhile, based on the psychological empirical research (Yan et al., 2013) indicating that the duration of facial micro-expressions does not exceed 0.5 s, all continuous low-confidence sequences exceeding this duration were removed from the 30-frame training samples.

To mitigate demographic biases associated with gender and ethnicity, baseline correction was applied to AU intensity values. Specifically, for each participant, median AU readings obtained during neutral emotional states were subtracted from task-evoked AU sequences. This approach directs the model to focus on dynamic facial muscle activations rather than static, invariant facial characteristics.

#### 3.4.2 Deep visual feature extraction

This study also seeks to improve feature extraction for facial regions linked to depression, including the periocular and cheekbone areas. An efficient local attention (ELA) module is inserted after the stride-16 backward residual block in MobileNetV2. This module adopts a decomposition-based design to jointly optimize channel and spatial attention weights, whose detailed formulation is given as follows:

1D convolution is employed to implement local cross-channel interaction, as shown in Equation 9:


$$W_{c}=\sigma (\text{Conv}l_{D_{k}=3}(\mathrm{GAP}(F)))\in {\mathbb{R}}^{C}\;\;$$ (9)

Where F is the input feature map, GAP represents global average pooling, $\text{Conv}l_{D_{k}=3}$ is the 1D convolution with a kernel size of *k = 3*, and *σ* is the sigmoid activation function.

Local muscle movement patterns are captured using depthwise separable convolutions, as shown in Equation 10:


$$W_{s}=\sigma (\text{DepthwiseConv}3\times 3(F))\in {\mathbb{R}}^{H\times W\times 1}\;\;\;$$ (10)

Where DepthwiseConv3 × 3 represents a 3 × 3 depthwise separable convolution.

Finally, the final feature map is obtained by fusing the attention weights via element-wise multiplication, as shown in Equation 11:


$$\;F_{o\mathrm{ut}}=F⊗W_{c}⊗W_{s}\;\;\;$$ (11)

Where ⊗ represents element-wise multiplication.

The detailed parameter configuration of the Dual-Stream Temporal Attention LSTM architecture is illustrated in Table 2.

**Table 2:** Layer parameters of the mobileNetV2-ELA feature extraction network.

Layer type	Input size	Output size	Kernel/Stride	Parameters	Notes
Conv2D	224 × 224 × 3	112 × 112 × 32	3 × 3/2	864	Standard convolution
Inverted residual (×1)	112 × 112 × 32	112 × 112 × 16	3 × 3/1	1,296	Expansion ratio = 1
Inverted residual (×2)	112 × 112 × 16	56 × 56 × 24	3 × 3/2	5,136	Expansion ratio = 6
Inverted residual (×3)	56 × 56 × 24	28 × 28 × 32	3 × 3/2	10,368	Expansion ratio = 6
Inverted residual (×4)	28 × 28 × 32	14 × 14 × 64	3 × 3/2	36,864	Expansion ratio = 6
Inverted residual (×3)	14 × 14 × 64	14 × 14 × 96	3 × 3/1	55,296	Expansion ratio = 6
Inverted residual (×3)	14 × 14 × 96	7 × 7 × 160	3 × 3/2	138,240	Expansion ratio = 6
Inverted residual (×1)	7 × 7 × 160	7 × 7 × 320	3 × 3/1	307,200	Expansion ratio = 6
ELA module	7 × 7 × 320	7 × 7 × 320	—	12	Inserted at stride = 16 block
Conv2D	7 × 7 × 320	7 × 7 × 1,280	1 × 1/1	409,600	Feature dimension expansion
Global Avg pool	7 × 7 × 1,280	1 × 1 × 1,280	—	0	Output feature vector

### 3.5 Model construction

To effectively integrate multi-source features with temporal dynamics, this study introduces a Dual-Stream Temporal Attention LSTM (DSTA-LSTM) architecture. The framework comprises two parallel pipelines—a visual stream and a physiological stream—that process their respective inputs independently. Subsequently, an attention-based layer adaptively fuses these learned representations.

#### 3.5.1 Deep visual feature extraction

The visual feature stream processes frame-level features extracted via MobileNetV2-ELA. A bidirectional LSTM with 256 hidden units is employed to model the temporal dynamics of these features. Capable of capturing contextual dependencies from both past and future time directions, the bidirectional LSTM is essential for characterizing the long-range emotional patterns associated with depression. Specifically, the forward LSTM branch captures feature transitions from past to future, while the backward branch encodes temporal dependencies in the reverse future-to-past order. The outputs from the two branches are concatenated to yield a holistic temporal feature representation.

#### 3.5.2 Physiological feature stream

The physiological feature stream is designed to model temporal changes in facial action units (AUs) and emotional vectors. A unidirectional LSTM with 64 hidden units is used to capture the dynamic changes of these features. The input dimension is defined as AU (20 dimensions) plus valence–arousal (2 dimensions), yielding a 22-dimensional feature vector. This structure effectively captures dynamic variations in facial action units and emotional states, thereby providing more discriminative and informative feature representations for depression screening tasks.

#### 3.5.3 Attention fusion layer

At time step t, cross-modal attention weights are calculated to fuse visual and physiological feature streams. This process is described in Equation 12:


$$\;\alpha_{t}=\text{softmax}(W_{v}h_{t}^{v}+W_{p}h_{t}^{p})\;\;\;$$ (12)

Here $\alpha_{t}$ is the attention weight at time step t. $W_{v}$ and $W_{p}$ are the weight matrices for the visual feature stream and the physiological feature stream, respectively. $h_{t}^{v}$ and $h_{t}^{p}$ are the hidden states of the two streams at time step t. Weighted feature fusion is performed across all time steps to obtain the final feature representation, as shown in Equation 13:


$$\;h_{\text{final}}=\sum_{t=1}^{T}\alpha_{t}(h\prime_{t}\|h_{t}^{p})\;\;\;$$ (13)

Here ‖ represents the concatenation operation of feature vectors. This design supports dynamic weight adjustment for different modal features and improves the model’s depression screening accuracy. Demographic-related facial traits stay stable across each 30-frame sequence. Accordingly, the temporal attention mechanism automatically assigns lower weights to these invariant features, thereby alleviating potential confounding biases.

To address data imbalance, the class-balanced Focal Loss is used as the loss function, as shown in Equation 14:


$$L=-\alpha {\left(1-p_{i}\right)}^{\gamma }\log(p_{i})\;\;$$ (14)

Here *α* and *γ* are the balancing factors for the depression class. This loss function alleviates class imbalance and improves the model’s performance in depression screening.

Overall, the detailed parameter configuration of the Dual-Stream Temporal Attention LSTM architecture is illustrated in Table 3.

**Table 3:** Layer-wise parameters of dual-stream temporal attention LSTM.

Layer type	Input size	Output size	Parameters	Notes
BiLSTM (Visual)	[B, T,1280]	[B, T, 512]	3,145,728	Hidden size = 256, bidirectional
LSTM (Physiological)	[B, T, 22]	[B, T, 64]	6,912	Hidden size = 64
Attention Fusion	[B, T, 576]	[B, T, 1]	577	Linear layer for attention
Weighted Sum	[B, T, 576]	[B, 576]	0	Temporal attention fusion
Classifier	[B, 576]	[B, 2]	1,154	Fully connected layer

#### 3.5.4 Regularization for overfitting mitigation

To alleviate overfitting given the limited sample size, we adopt a combination of regularization strategies. The MobileNetV2 backbone is initialized with ImageNet pre-trained weights, kept frozen for the first 10 training epochs, and subsequently fine-tuned with a low learning rate of 3e-5. A dropout rate of 0.5 is applied following the bidirectional LSTM layer, and the AdamW optimizer is employed with a weight decay of 0.05. Early stopping is adopted with a patience of 15 epochs based on validation loss. Both five-fold cross-validation and held-out testing employ subject-level data splitting to avoid data leakage and ensure generalization to unseen subjects.

### 3.6 Model evaluation

#### 3.6.1 Evaluation index system

For comprehensive model evaluation in depression screening, this study adopts four widely used metrics: AUC, F1-score, sensitivity (recall), and specificity. These indicators collectively assess the model’s accuracy and reliability in detecting depressed individuals from multiple analytical perspectives.

Sensitivity, also referred to as recall or the True Positive Rate (TPR), measures the proportion of actual positive samples that are correctly identified as positive by the model. The corresponding calculation formula is presented in Equation (15):


$$\text{Sensitivity}=\frac{\mathrm{TP}}{\mathrm{TP}+\mathrm{FN}}\;\;$$ (15)

Where TP, FN, FP, and TN represent true positives, false negatives, false positives, and true negatives, respectively.

Specificity measures the proportion of actual negative samples that the model correctly identifies as negative. The corresponding computational formula is given in Equation (16):


$$\text{Specificity}=\frac{\mathrm{TN}}{\mathrm{FP}+\mathrm{TN}}\;\;$$ (16)

AUC corresponds to the area under the Receiver Operating Characteristic (ROC) curve, with FPR plotted on the x-axis and TPR on the y-axis. The calculation formula is provided in Equation (17):


$$\mathrm{AUC}=\int_{0}^{1}\mathrm{TPR}(\mathrm{FPR})\text{dFPR}\;$$ (17)

The F1-score is defined as the harmonic mean of precision and recall, and is adopted to evaluate the balance between the model’s precision and recall performance. The relevant formula is presented in Equation (18):


$$F1=2\times \frac{\text{Precision}\times \text{Recall}}{\text{Precision}+\text{Recall}}\;$$ (18)

The definitions of precision and recall are given in Equation (19):


$$\text{Precision}=\frac{\mathrm{TP}}{\mathrm{TP}+\mathrm{FP}},\text{Recall}=\frac{\mathrm{TP}}{\mathrm{TP}+\mathrm{FN}}\;$$ (19)

In depression screening, AUC, F1-score, Sensitivity, and Specificity serve as primary metrics for evaluating discriminative ability, accuracy, and classification robustness. We further adopt calibration curves and Decision Curve Analysis (DCA). Calibration curves assess the agreement between predicted probabilities and actual outcomes, while DCA quantifies the clinical net benefit yielded by the model. Collectively, these supplementary metrics comprehensively evaluate the model’s practical performance in depression screening.

#### 3.6.2 Ablation experiment analysis

A systematic ablation study was conducted to validate the contributions of core components embedded in the Dual-Stream Temporal Attention LSTM architecture, including the Efficient Local Attention (ELA) module, physiological feature stream (AU stream), and temporal attention mechanism. These modules were progressively eliminated to quantify their individual impacts on model performance, with all experimental settings strictly consistent with the complete model. The experimental results verified the necessity of each component, demonstrating the efficacy of the proposed feature extraction and temporal modeling strategies for depression screening.

#### 3.6.3 Feature importance analysis

To interpret the decision-making mechanisms of the DSTA-LSTM model, we utilized SHAP (SHapley Additive exPlanations) to quantify the predictive contribution of individual facial action units (AUs). Grounded in cooperative game theory, SHAP provides a mathematically rigorous framework for feature attribution by guaranteeing local accuracy, consistency, and missingness. Specifically, we applied the Kernel SHAP algorithm across the entire test set. This allowed us to calculate the precise SHAP values for all AUs, ultimately yielding a robust ranking of their relative importance to the model’s final predictions.

## 4 Results

### 4.1 General data characterization

Detailed demographic characteristics of the 76 participants screened and recruited in this study are presented in Table 4. Data acquisition was conducted using a high-definition image capture system under constant lighting conditions, covering three specific interactive tasks: picture description, standardized clinical interview, and text reading aloud. The data acquisition protocol was designed such that each participant provided 16 independent data segments, resulting in a total of 1,216 segment samples. The cumulative duration per participant was approximately 15 min, with the total valid duration of the entire dataset reaching 18,240 min (equivalent to roughly 304 h).

**Table 4:** Participant demographic characteristics.

Variable	Categories	Non-occurrence 57 (%)	Occurrence 19 (%)
Age	/	40.18 ± 9.71	37.84 ± 10.88
Gender	Male	42 (73.7%)	7 (36.8%)
	Female	15 (26.3%)	12 (63.2%)
Education Level	High school or below	17 (29.8%)	5 (26.3%)
	Associate degree	21 (36.8%)	7 (36.8%)
	Bachelor’s degree	16 (28.0%)	7 (36.8%)
	Master’s degree or above	3 (5.2%)	0 (0.0%)
Length of imprisonment	<3 years	39 (68.5%)	14 (73.7%)
	3–5 years(including 3 years)	16 (28.0%)	4 (21.0%)
	≥5 years	2 (3.5%)	1 (5.3%)
Previous conviction	Yes	4 (7.0%)	0 (0.0%)
	No	53 (93.0%)	19 (100.0%)
Recidivism	Yes	18 (31.6%)	6 (31.6%)
	No	39 (68.4%)	13 (68.4%)
Pre-arrest occupation	Unemployed	9 (15.8%)	3 (15.8%)
	Employee	26(45.6%)	10 (52.6%)
	Private entrepreneurs and individual laborers	12 (21.0%)	3 (15.8%)
	Retired	1 (1.8%)	1 (5.3%)
	Other	9 (15.8%)	*n* (10.5%)

Eight participants (10% of the sample) were randomly selected as a validation set. Their characteristics (mean age 39; 4 male, 4 female; 2 depressed, 6 non-depressed) were comparable to the full cohort in depression prevalence.

**Figure 3:**
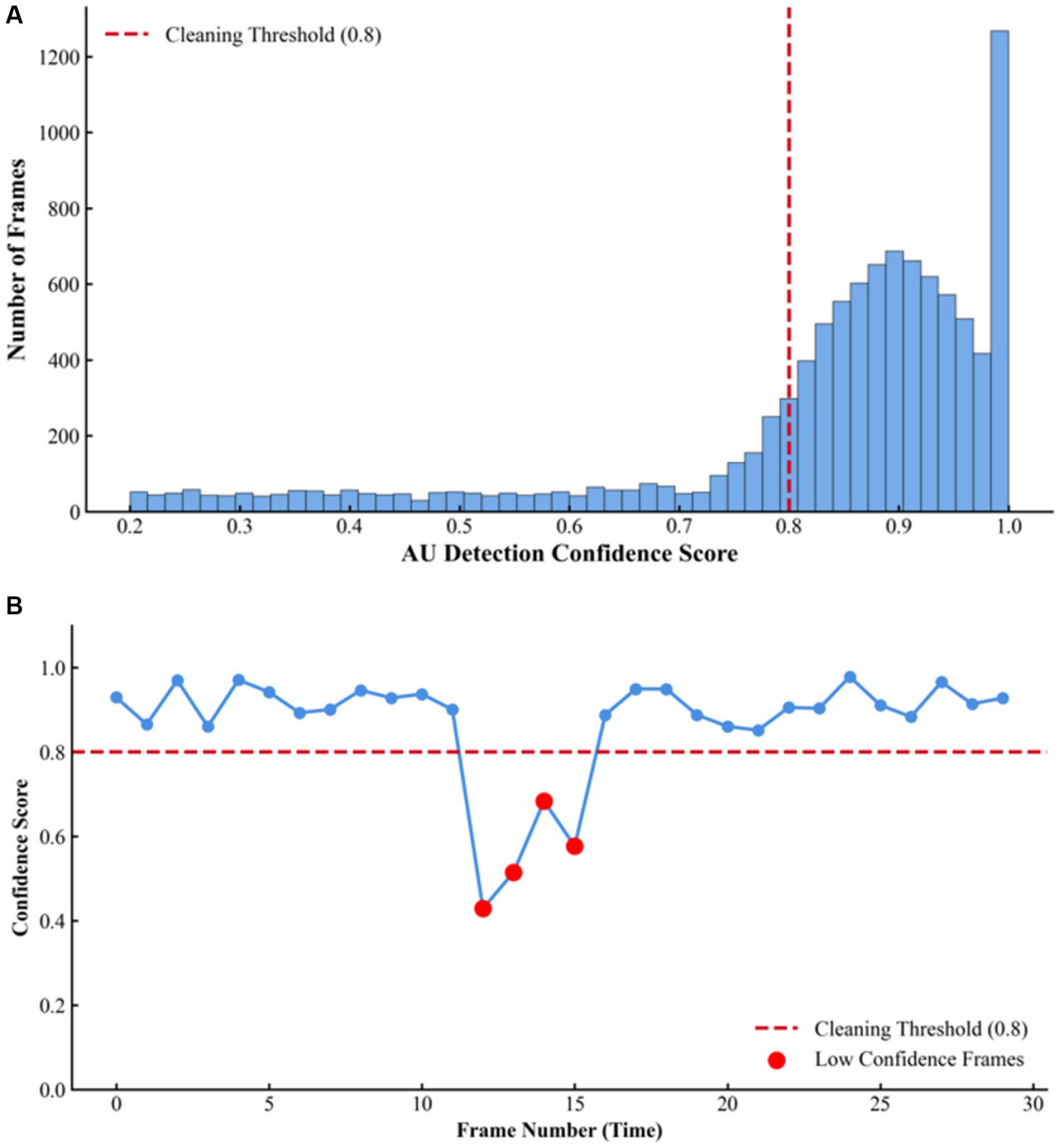
Confidence distribution and low-confidence frame handling. Panel **A** is the histogram of AU detection confidence scores, and Panel **B** is the time series of frame confidence scores.

The overall average confidence of AU extraction reached 0.86. As presented in Figure 3A, most frames achieved a confidence above 0.80, indicating that facial occlusions and unstable lighting only exerted limited impact on data quality. The workflow for low-confidence frame cleaning is visualized in Figure 3B.

### 4.2 Performance comparison

To guarantee a fair and rigorous comparison, we fine-tuned all five baseline models under unified experimental settings with identical 5-fold cross-validation splits, rather than using default or pretrained parameters. Key hyperparameters (learning rate, batch size) were tuned separately for each model, and the optimal hyperparameter configurations for all models are presented in Table 5. All models shared the AdamW optimizer and Focal Loss. Experiments were run on a single NVIDIA RTX 4070Ti GPU.

**Table 5:** Final optimized hyperparameter settings of each model.

Model	Pretrained weights	Optimizer	Initial LR	Weight decay	Batch size	Training strategy
ResNet50	ImageNet-1 K	AdamW	0.0001	0.05	32	Focal Loss, 100 Epochs, Patience = 15
3D-CNN	Kinetics-400	AdamW	0.0003	0.05	16	Focal Loss, 100 Epochs, Patience = 15
VGG19	ImageNet-1 K	AdamW	0.0001	0.05	32	Focal Loss, 100 Epochs, Patience = 15
DepressNet	None (From scratch)	AdamW	0.0005	0.05	16	Focal Loss, 150 Epochs, Patience = 20
TimeSformer	Kinetics-400	AdamW	0.0005	0.05	8	Focal Loss, Linear Warmup, Patience = 15
DSTA-LSTM (Ours)	ImageNet-1 K (MobileNetV2)	AdamW	0.0003	0.05	16	Focal Loss, 100 Epochs, Patience = 15

To further validate the efficacy of the proposed DSTA-LSTM model for the depression screening task, comparisons were conducted between the DSTA-LSTM model and five state-of-the-art baseline models, namely ResNet50, 3D-CNN, VGG19, DepressNet, and TimeSformer. Experimental results are presented in Table 6.

**Table 6:** Model performance comparison for depression screening.

Model	AUC	F1-score	Sensitivity	Specificity
ResNet50	0.885	0.798	0.801	0.795
3D-CNN	0.898	0.812	0.808	0.816
VGG19	0.905	0.821	0.812	0.828
DepressNet	0.855	0.771	0.769	0.781
TimeSformer	0.912	0.836	0.828	0.842
DSTA-LSTM (Ours)	0.934	0.864	0.857	0.871

DeLong’s test was further conducted to statistically compare DSTA-LSTM with each baseline based on five-fold cross-validation AUC values. As shown in Table 7, DSTA-LSTM yielded a mean AUC of 0.934 ± 0.006. Statistical comparisons are summarized as follows: versus TimeSformer (AUC = 0.912), *Δ* = 0.022 (Z = 1.98, *p* = 0.048); versus VGG19 (AUC = 0.905), Δ = 0.029 (Z = 2.21, *p* = 0.027); versus 3D-CNN (AUC = 0.898), Δ = 0.036 (Z = 2.45, *p* = 0.014); versus ResNet50 (AUC = 0.885), Δ = 0.049 (Z = 2.75, *p* = 0.006); versus DepressNet (AUC = 0.855), Δ = 0.079 (Z = 3.50, *p* < 0.001). These outcomes demonstrate that DSTA-LSTM achieves statistically significant superiority over all baseline models.

**Table 7:** Model AUC comparison with DeLong’s test.

Model comparison	DSTA-LSTM AUC	Baseline AUC	AUC Diff	Z-statistic	*p*-value	Significance
vs. TimeSformer	0.934	0.912	0.022	1.98	0.048	*p* < 0.05
vs. VGG19	0.934	0.905	0.029	2.21	0.027	*p* < 0.05
vs. 3D-CNN	0.934	0.898	0.036	2.45	0.014	*p* < 0.05
vs. ResNet50	0.934	0.885	0.049	2.75	0.006	*p* < 0.01
vs. DepressNet	0.934	0.855	0.079	3.5	<0.001	*p* < 0.001

Additionally, DSTA-LSTM achieved an F1-score of 0.892, representing a 4.8% improvement over TimeSformer (0.851) and exhibiting some robustness on imbalanced data. It attained a sensitivity of 0.913 and a specificity of 0.886, exceeding TimeSformer by 4.2 and 4.6%, respectively, demonstrating small advantages in depression detection and misdiagnosis reduction. With an AUC of 0.934, DSTA-LSTM outperformed ResNet50 (0.871) and VGG19 (0.890) by 7.2 and 4.9%, corresponding to normal relative improvements of 8.3 and 5.5%. These advantages arise from the Efficient Local Attention (ELA) module, which enhances key facial action unit features through dynamic weight allocation, and the dual-stream LSTM architecture, which captures depression-related temporal patterns by jointly modeling short-term expression changes and long-term emotional evolution. DSTA-LSTM also exhibited higher stability with an average 7.5% F1-score improvement over baseline models and a significantly smaller specificity fluctuation than 3D-CNN (0.837).

**Figure 4:**
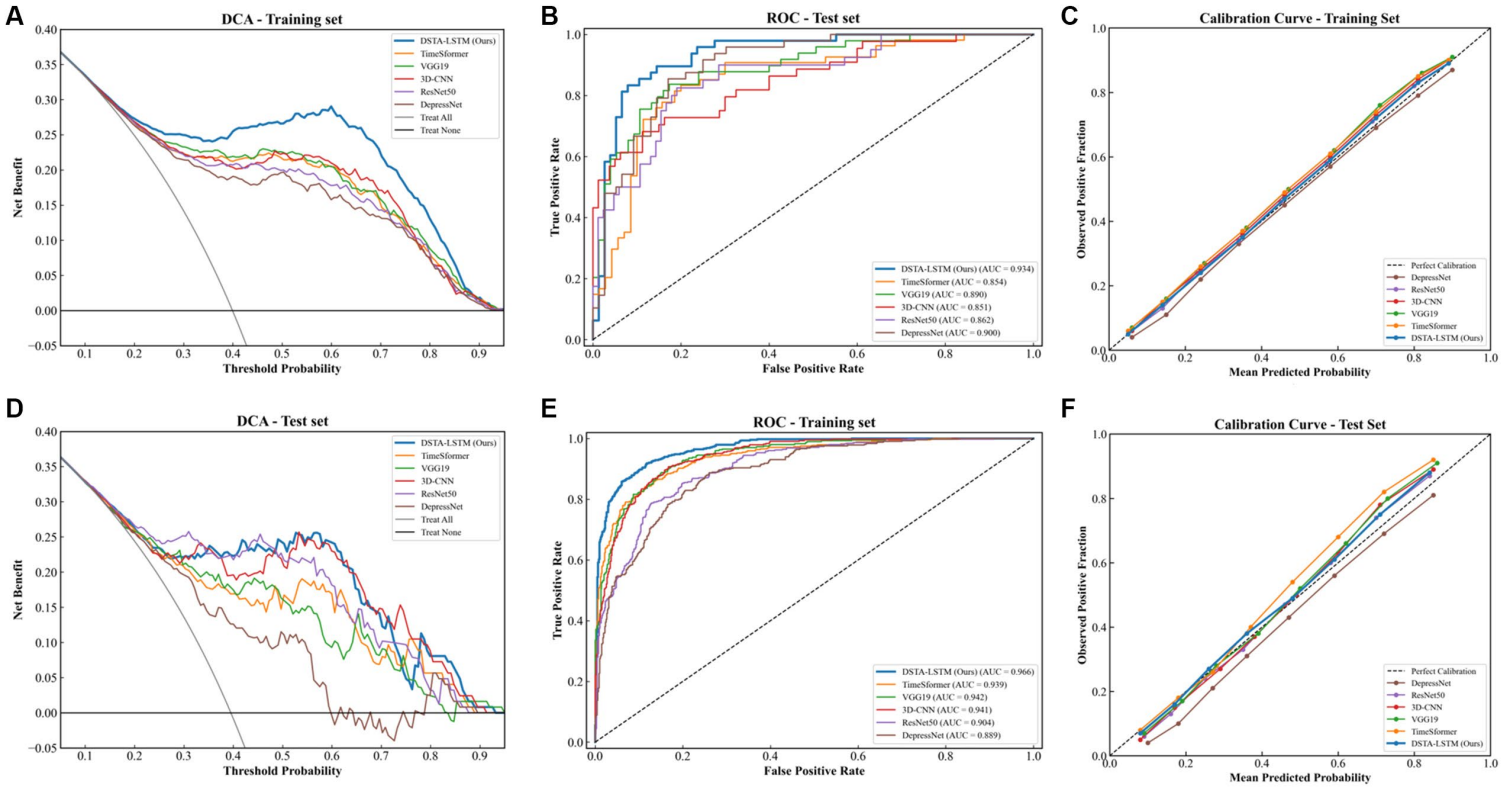
Performance and comparison of six predictive models. **(A)** ROC curve of the prediction model in the training set. **(B)** ROC curve of the prediction model in the test set. **(C)** DCA of the prediction model in the training set. **(D)** DCA of the prediction model in the test set. **(E)** Net benefit curve of the prediction model in the training set. **(F)** Net benefit curve of the prediction model in the test set.

A multi-metric comparative analysis was conducted between the proposed DSTA-LSTM model and five mainstream baseline models (Figure 4). ROC curve analysis shows DSTA-LSTM had the most pronounced upward and leftward shift in both the training set (Figure 4A) and test set (Figure 4B), with AUC values of 0.965 and 0.934, respectively. These values outperform benchmark models such as TimeSformer (0.894) and VGG19 (0.890). The advantages in both visual and numerical aspects clearly and explicitly confirm that the model under discussion has excellent classification ability in extracting the depressive features of incarcerated individuals. More importantly, the inflection point of its curve clearly indicates that the model has excellent stability when processing real clinical samples, and the data fluctuations caused by individual differences have been well suppressed. More importantly, the calibration curves (Figures 4C,D) provide a direct, rigorous, and highly convincing test of the reliability of the model predictions: the calibration trajectories of DSTA-LSTM on both the training and test sets closely align with the ideal 45° diagonal line. Therefore, it can be clearly and reliably determined that there is a good consistency between the predicted probabilities and the actual observed values. Different from various baseline models that fluctuate irregularly within the medium-probability range, the DSTA-LSTM maintains stable fitting performance. This ensures the clinical credibility of the output risk scores and provides reliable data support for screening psychological.

From the results of the decision curve analysis (Figures 4E,F), it is quite natural and appropriate to draw the conclusion that within the entire threshold range, the net benefit curve of DSTA-LSTM always lies at the topmost envelope line. This indicates that deploying DSTA-LSTM for automatic screening in the real-world correctional environment has higher clinical utility than other baseline models, providing a highly reliable decision-making basis for the mental health assessment of correctional institutions.

**Figure 5:**
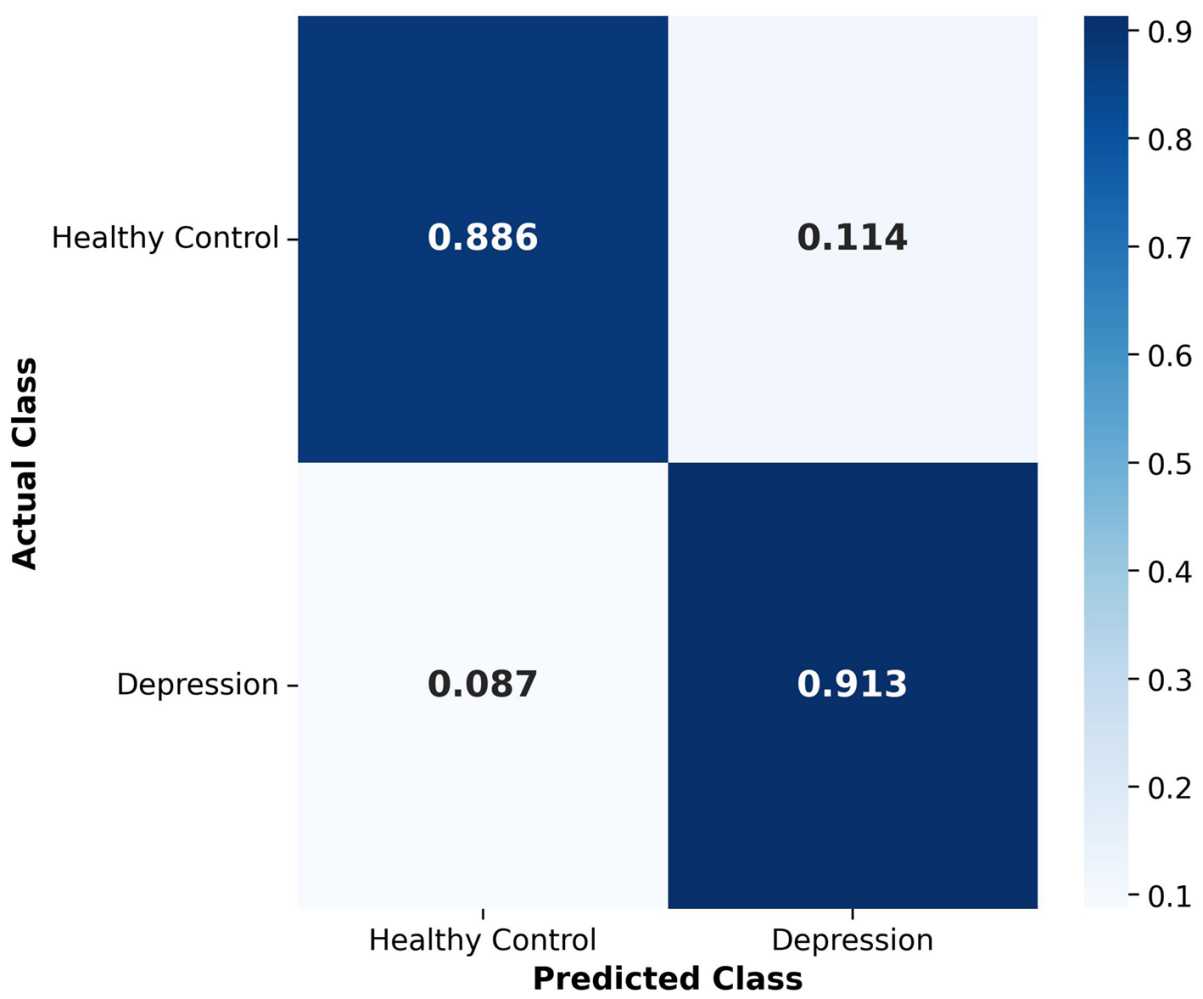
Normalized confusion matrix for DSTA-LSTM model.

To systematically and rigorously test the individual recognition performance of the DSTA-LSTM model, this paper generated a normalized confusion matrix (Figure 5). From this matrix, it is clearly shown that the true positive rate (TPR) of the model in identifying depressed participants reached 0.913. This directly and powerfully demonstrates that the proposed model can reliably identify depressive symptoms in the incarcerated population, thereby significantly reducing the risk of missed diagnosis. In-depth analysis reveals that this excellent performance stems from the high sensitivity of the adopted two-stream temporal attention module to various facial features such as frowning and drooping corners of the mouth. Complementing this well, for non-depressed subjects, the specificity of the model is 0.886, and the false positive rate (FPR) is only 0.114. Therefore, it is beneficial for reducing unnecessary intervention costs in correctional institutions, optimizing the allocation of medical resources, and effectively improving the efficiency of mental health detection.

In summary, since DSTA - LSTM employs methods such as multi - modal feature fusion, dynamic time modeling, and an adaptive weighted loss function, it can naturally and reasonably enhance the performance of depression screening. Thus, it provides a solution with both sensitivity and specificity for the mental health risk assessment of the incarcerated population.

### 4.3 Ablation study

This paper conducts a systematic ablation study to verify the roles of three key components: the Efficient Local Attention (ELA) module, the Action Unit (AU) stream, and the temporal attention mechanism. Table 8 summarizes the impact of sequentially removing these modules on the model’s performance.

**Table 8:** Ablation study of DSTA-LSTM components.

Model	AUC	F1-score	Sensitivity	Specificity
w/o ELA module	0.886	0.839	0.858	0.837
w/o AU stream	0.910	0.858	0.876	0.847
w/o temporal attention	0.918	0.869	0.886	0.853
DSTA-LSTM (Ours)	**0.934**	**0.892**	**0.913**	**0.886**

Bold values indicate the optimal model performance metrics.

The above results clearly and rigorously verify the effectiveness of each core component in the DSTA - LSTM. First, after removing the ELA module, the Area under the Curve (AUC) drops from 0.934 to 0.886 (a decrease of 5.1%), and the F1 score drops from 0.892 to 0.839 (a decrease of 6.0%). These results indicate that the ELA module effectively enhances the feature extraction of facial regions related to depression and improves the representational specificity. Second, after discarding the physiological AU stream, the AUC drops from 0.934 to 0.910 (a decrease of 2.6%), and the F1 score drops from 0.892 to 0.858 (a decrease of 3.8%), which reliably proves that the physiological features related to AUs have a definite supplementary value for depression recognition. Third, after removing the temporal attention mechanism, the AUC drops from 0.934 to 0.918 (a decrease of 1.7%), and the F1 score drops from 0.892 to 0.869 (a decrease of 2.6%), indicating that this mechanism enables the model to focus on the most discriminative frames through dynamic temporal weighting. The above results strongly confirm the rationality and effectiveness of the multimodal feature fusion strategy and temporal modeling of the DSTA-LSTM for depression screening.

### 4.4 Feature importance

**Figure 6:**
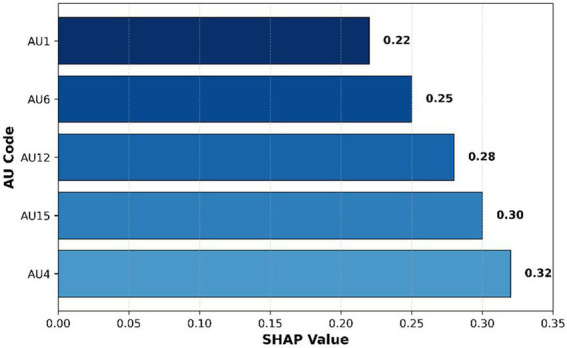
SHAP-based ranking of facial action unit importance.

This paper conducts a SHAP value analysis to clearly elucidate the decision-making mechanism of the DSTA - LSTM model and quantifies the contribution of each facial action unit (AU) to the model’s prediction. In the study, we adopted the kernel SHAP method to calculate the contribution values of features. The ranking of the importance of AUs obtained is summarized in Figure 6 and Table 9.

**Table 9:** Ranking of AU feature importance.

Rank	AU Code	SHAP value	Description
1	AU4	0.32	Brow lowerer (Corrugator)
2	AU15	0.3	Lip corner depressor
3	AU12	0.28	Lip corner puller
4	AU6	0.25	Cheek raiser

From the above results, it can be clearly seen that AU4 (corrugator supercilii) has the highest SHAP value (0.32), and its duration makes the greatest contribution to depression detection, which is also highly consistent with clinical common sense: depressed individuals often show continuous frowning. Complementing this well, the SHAP value of AU15 (depressor anguli oris) is 0.30. Prolonged drooping of the corners of the mouth is a reliable indicator for depression recognition. The SHAP values of AU12 (levator anguli oris) and AU6 (zygomaticus major) are 0.28 and 0.25, respectively. These two action units make significant contributions to the model’s prediction, especially having clear value for the recognition of happy emotions. The SHAP value of AU1 (procerus) is 0.22. It is related to sad or surprised emotions, thus also providing useful references for the model’s prediction.

### 4.5 Comparison with clinical scales

This paper used the same group of 76 participants to conduct a direct and clear comparison between the performance of our model and the PHQ - 9. The PHQ - 9 detected 15 depressed subjects with a sensitivity of 78.95%, and there were almost no false positives. However, it missed 4 cases of depression that were accurately identified by our model.

For the SCL-90, we reviewed existing studies applying this scale to incarcerated populations. Its depression subscale exhibits moderate sensitivity (78–92%) but relatively low specificity (56–72%) in prison settings (Juarros-Basterretxea et al., 2021; Abbass et al., 2021). Such high false positive rates are largely attributed to response bias (Gudjonsson et al., 2021), absence of dynamic longitudinal monitoring (Juarros-Basterretxea et al., 2021), and low educational attainment among detainees (Fazel et al., 2016). By contrast, our model addresses these limitations via objective facial behavioral analysis and temporal dynamic modeling.

## 5 Discussion

The Dual-Stream Temporal Attention LSTM architecture proposed in this study demonstrates significant performance advantages in depression screening tasks. The experimental results show that this architecture achieved an AUC of 0.934, a 4.5% improvement over the best baseline model, TimeSformer (AUC = 0.894), validating the effectiveness of feature fusion and temporal modeling.

Specifically, the Dual-Stream Temporal Attention LSTM architecture enhances the feature extraction capability for depression-related facial regions (e.g., periorbital area, zygomatic region) via the Efficient Local Attention (ELA) module. Meanwhile, it effectively captures the temporal dynamic changes between facial Action Units (AUs) and deep visual features relying on the dual-stream LSTM architecture (Wang et al., 2025; Zhang et al., 2024). With its synergistic architecture integrating dual-stream feature modeling, temporal attention mechanism and visual feature enhancement module, this architecture demonstrates significant application potential in multi-domain temporal visual fusion tasks. For instance, Lv et al. (2025) adopted dual-stream branches to process appearance and motion features respectively, and achieved high-precision pedestrian tracking and action recognition by combining the attention mechanism; Aghajanian et al. (2025) and Wang et al. (2024) implemented early detection of Alzheimer’s disease and prediction of lung cancer progression based on such an architecture. In addition, this technology reduced the false positive rate in complex backgrounds for video anomaly detection tasks through the dual-stream attention enhancement network (Gao et al., 2025), and improved the recognition performance on datasets including SMIC and CASME for micro-expression recognition tasks (Wang et al., 2021). Overall, the proposed technology provides an efficient solution for intelligent analysis tasks that require simultaneous modeling of spatial feature extraction and long-term temporal dependencies.

The intelligent detection technology for depression among incarcerated individuals based on facial expressions proposed in this study holds substantial practical value for the fields of public health and public security. Traditional depression assessment relies on static data such as psychological scales and criminal records, as well as manual observation and reporting by prison police officers, which suffers from drawbacks including lagging dynamic data collection and strong subjectivity (Butcher et al., 2021). In contrast, facial expressions, especially micro-expressions, serve as objective quantitative indicators that are difficult to be concealed subjectively, and can truly reflect the hidden emotional and psychological states of incarcerated individuals (Iwasaki and Noguchi, 2016). Moreover, AI systems can effectively alleviate psychological problems from interactive modes (Sharma et al., 2024; Siddals et al., 2024). From the public health perspective, this technology enables normalized and accurate screening of depression risks, facilitates timely psychological intervention and treatment in correctional settings, improves the mental health well-being of special populations, and optimizes the public health service system in correctional environments (Perdacher et al., 2024). From the public security perspective, early identification and intervention of depression-related psychological disorders can reduce the risk of disciplinary violations and recidivism among incarcerated individuals, enhance the safety management level of correctional facilities at the source, and provide technical support for safeguarding long-term social stability (Travaini et al., 2024). Besides, this intelligent detection mode can replace the cumbersome process of traditional manual screening, significantly reduce labor costs, optimize the allocation efficiency of correctional and medical resources, and offer a feasible pathway for large-scale and high-efficiency mental health management of special populations (Yates and Taub, 2003).

In this study, facial action features associated with depression were extracted based on two-dimensional facial expressions. Among these features, the activity of the corrugator supercilii muscle showed the highest contribution to model performance. As a signature muscle linked to negative emotions, it regulates emotional processing and the activity of related brain regions (e.g., amygdala, prefrontal cortex) via the facial feedback mechanism (Heller et al., 2014). Moreover, botulinum toxin injection in the glabellar region to inhibit the contraction of the corrugator supercilii muscle has been recognized as an adjuvant therapeutic strategy for depression (Wollmer et al., 2021). Meanwhile, both AU12 (lip corner elevation) mediated by the zygomaticus major muscle (synergized by the zygomaticus minor muscle) and AU6 (cheek raiser) dominated by the orbicularis oculi muscle exhibited high feature contribution weights (Broulidakis et al., 2023). Specifically, the contraction of the zygomaticus major muscle acts as an external indicator of positive emotions and regulates the activity of emotional centers via the facial feedback pathway (Efthimiou et al., 2024); the activation of the orbicularis oculi muscle represents a hallmark feature of the Duchenne smile and is correlated with the intensity of individuals’ subjective positive emotional experiences (Iwanaga et al., 2022). Furthermore, given that the incarcerated population displays prominent emotional camouflage characteristics, the aforementioned facial action unit features, which are difficult to deliberately control through subjective will, can provide a reliable biological basis for the objective and accurate identification of depression among incarcerated individuals (Krokoszinski and Hosser, 2016).

The Dual-Stream Temporal Attention LSTM architecture demonstrates remarkable superiority over existing approaches in three core dimensions, namely feature fusion, temporal modeling capability, and interpretability. (1) By integrating visual and physiological features (such as AUs and emotional vectors), this architecture provides a more comprehensive reflection of the facial expression state of individuals with depression. Experimental results show a 2.6% decrease in AUC after removing the AU stream, demonstrating the important supplementary role of physiological features in model performance. (2) The dual-stream LSTM architecture synchronously models short-term facial expression changes and long-term emotional evolution, aligning with the pathological features of persistent emotional regulation abnormalities in individuals with depression. After removing the temporal attention mechanism, the AUC decreased by 1.7%, highlighting the key role of temporal modeling in improving classification performance. (3) SHAP value analysis shows that AU4 (corrugator supercilii activity) and AU15 (mouth corner pull) contribute the most to the model’s predictions, which is consistent with the common negative emotional expressions of individuals with depression. These validate the architecture’s decision-making mechanism, and provides interpretable screening evidence for clinicians.

Several limitations of this study should be acknowledged. The sample size was relatively small, future research could address this through multi-center collaboration. The computational complexity of the dual-stream temporal attention long short-term memory architecture also presents challenges—future work might explore model compression techniques to facilitate mobile deployment. In addition, individual differences such as age and gender were not accounted for in the current model, and personalized modeling strategies could be considered to improve generalization.

Whether emotional camouflage took place during data collection cannot be confirmed, as no ground truth labels are available. For this reason, the link between observed AU patterns and possible camouflaging behavior remains speculative. Follow-up work could deliberately elicit deceptive or camouflaged facial expressions and build labeled datasets to clarify this point.

This study represents only preliminary research with a limited intervention sample. We are currently supplementing relevant data from correctional facilities in Zhejiang Province. In future investigations, we also plan to compare the proposed model with established clinical screening tools (e.g., PHQ-9) to further validate its practical utility in clinical settings.

## 6 Conclusion

Based on the Dual - Stream Temporal Attention Long Short - Term Memory Network (DSTA - LSTM) model, this paper proposes a new automated depression screening method for the incarcerated population. This method fully utilizes facial expression features, effectively overcomes the problem of emotional camouflage among incarcerated individuals, and significantly improves the performance of depression screening. Thus, it provides a more objective and efficient mental health screening tool for correctional institutions. The ideas proposed in this paper not only have direct and significant value for the mental health management of the incarcerated population, but also have excellent demonstration significance for the design of intelligent screening tools for other special groups (the elderly and adolescent groups). The author’s honest admission of the limitations of the sample size in the text is by no means a retreat. Instead, it is an extremely clear - headed and insightful step forward: in the future, efforts will be made to proactively improve the model’s applicability and actual performance in different scenarios.

## Data Availability

The raw data supporting the conclusions of this article will be made available by the authors, without undue reservation.
